# Gut–Brain Axis in Focus: Polyphenols, Microbiota, and Their Influence on α-Synuclein in Parkinson’s Disease

**DOI:** 10.3390/nu16132041

**Published:** 2024-06-27

**Authors:** Elizabeth Riegelman, Kathy S. Xue, Jia-Sheng Wang, Lili Tang

**Affiliations:** Department of Environmental Health Science, University of Georgia, Athens, GA 30602, USA; eriegelman@uga.edu (E.R.); ksxue@uga.edu (K.S.X.); jswang@uga.edu (J.-S.W.)

**Keywords:** polyphenols, α-synuclein, gut–brain axis, Parkinsons’s disease, gut microbiota

## Abstract

With the recognition of the importance of the gut–brain axis in Parkinson’s disease (PD) etiology, there is increased interest in developing therapeutic strategies that target α-synuclein, the hallmark abhorrent protein of PD pathogenesis, which may originate in the gut. Research has demonstrated that inhibiting the aggregation, oligomerization, and fibrillation of α-synuclein are key strategies for disease modification. Polyphenols, which are rich in fruits and vegetables, are drawing attention for their potential role in this context. In this paper, we reviewed how polyphenols influence the composition and functional capabilities of the gut microbiota and how the resulting microbial metabolites of polyphenols may potentially enhance the modulation of α-synuclein aggregation. Understanding the interaction between polyphenols and gut microbiota and identifying which specific microbes may enhance the efficacy of polyphenols is crucial for developing therapeutic strategies and precision nutrition based on the microbiome.

## 1. Introduction

Parkinson’s disease (PD) is the second most common neurodegenerative disease, after Alzheimer’s, affecting approximately 2–3% of the population more than 60 years of age and up to 5% of the population over 85 years of age [1,2]. It is estimated that there are 1.5 million people in the United States living with PD, and 60,000 Americans are diagnosed with this disease annually [3]. PD is a progressive neurological disorder that primarily affects motor function, leading to symptoms such as tremors, stiffness, slowness of movement, and impaired balance and coordination [4,5]. One of the hallmark pathologies of PD is the accumulation of alpha-synuclein (α-synuclein), a presynaptic neuronal protein that aggregates into insoluble fibrils, forming Lewy bodies (LBs) and Lewy neurites in the brain [6]. Although the exact function of α-synuclein remains partially understood, it has been linked to a range of neuronal functions, including neurotransmitter release, the modulation of a variety of enzymes and transporters, the dynamics of presynaptic vesicles, and the support of neuronal plasticity [7]. Its abnormal accumulation contributes to neuronal death and the subsequent manifestation of PD symptoms [8].

The pathogenesis of PD has not yet been fully elucidated. While the majority of earlier PD studies focused solely on brain pathologies, the gastrointestinal (GI) system is now recognized as a pivotal participant in the pathogenesis of PD [9,10,11,12,13]. Recent research has increasingly focused on the gut–brain axis, a complex bidirectional communication system that links the enteric nervous system (ENS) of the GI tract with the central nervous system (CNS). This axis not only plays a critical role in maintaining GI homeostasis but also appears to be involved in the pathogenesis of several neurological disorders, including PD. The emerging evidence suggests that α-synuclein pathology may originate in the gut and then spread to the brain via the vagus nerve, a concept supported by observations of GI abnormalities in PD patients years before the onset of motor symptoms. Moreover, alterations in the gut microbiota composition have been observed in PD patients, indicating a potential role of gut dysbiosis in the disease’s progression.

Currently, there is no existing cure for PD, and drugs that are presently used in clinics only provide symptomatic treatment rather than preventing or slowing the pathogenic progression of neurodegeneration [14]. In addition, some of these drugs present many side effects in patients [15]. Therefore, there is an urgent need to develop novel therapeutic agents with lower side effects and a broader spectrum of targets to not only treat the symptoms but also potentially prevent or slow the pathogenic progression of PD. Given the interaction between the gut microbiota and the CNS, dietary interventions that modulate the gut microbiome have garnered interest as a potential therapeutic strategy for PD. Polyphenols, a diverse group of phytochemicals found in fruits, vegetables, tea, wine, and certain herbs, have garnered attention in this context. These compounds are known for their antioxidant and anti-inflammatory properties and have been shown to influence the composition and function of the gut microbiota. Importantly, polyphenols may also inhibit the aggregation of α-synuclein, offering a dual mechanism by which they could mitigate PD pathology. By modulating the gut microbial community, dietary polyphenols may reduce intestinal inflammation, enhance gut barrier function, and produce metabolites that could potentially inhibit the aggregation of α-synuclein. Therefore, understanding the mechanisms underlying the interactions between dietary polyphenols, the gut–brain axis, and α-synuclein pathology could open new avenues for preventing or slowing the progression of PD, highlighting the significance of diet and gut microbiota in the pathogenesis of neurodegenerative diseases. This review aims to integrate the current knowledge and recent discoveries to shed light on the complex interactions between dietary polyphenols, gut microbiota, and α-synuclein aggregation, offering insights into potential novel therapeutic strategies for PD that leverage the gut–brain axis.

## 2. α-Synuclein: A Key Player in Parkinson’s Disease Pathology

α-synuclein is a protein comprising 140 amino acids encoded by the *SNCA* gene, which normally exists in naturally occurring monomers. Under pathological conditions, natively unfolded monomers can undergo self-aggregation, forming pathological oligomers that further mature into fibrils [16]. The accumulation of α-synuclein aggregates (particularly into oligomers and fibrils) disrupts normal cellular processes, causes mitochondrial dysfunction, triggers inflammation, and leads to neuronal death. Therefore, treatments that target α-synuclein oligomers and/or fibrils may reduce neurodegeneration, which is a promising therapeutic strategy for PD [17].

### 2.1. α-Synuclein Structure and Physiological Function

α-synuclein is a relatively small protein within the synuclein family, which also includes β-synuclein and γ-synuclein. With a molecular weight of approximately 14 kDa, α-synuclein is predominantly located at the presynaptic terminals in the CNS and accounts for about 1% of all cytosolic proteins in the brain [7,18].

**Figure 1:**
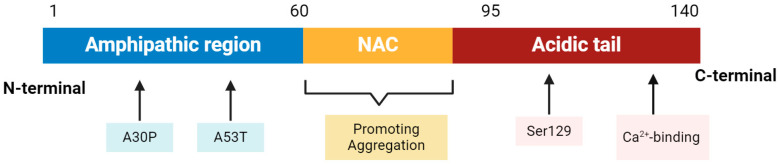
Scheme of α-synuclein regions with the number of corresponding residues.

A schematic figure about the structure of α-synuclein is shown in Figure 1. The protein can be divided into three distinct domains: The N-terminal amphipathic domain, which contains the evolutionary conserved KTEGV motifs and the main mutations associated with familial PD, such as A53T and A30p [19]. This region is responsible for membrane binding as well. Additionally, there is a central hydrophobic segment known as the non-amyloid component (NAC), which confers the potential for a β-pleated sheet, and a highly negatively charged carboxyl tail at the C-terminal end, which contains a Ca^2+^-binding site and the main phosphorylation site at Ser129, modulating α-synuclein aggregation. The hydrophobic NAC region, in particular, is critical for the protein’s propensity to adopt β-sheet configurations that can self-assemble into fibrillar aggregates, forming the core of LBs, a pathological hallmark found in PD brains [16]. Once α-synuclein fibrils are established in a neuron, they can act as a guide for the aggregation of endogenous α-synuclein protein [20], further initiating PD progression.

Although the main function of α-synuclein in both the central and peripheral nervous systems remains unclear, it has been linked to a range of neuronal functions, including neurotransmitter release, the modulation of a variety of enzymes and transporters, the dynamics of presynaptic vesicles, and the support of neuronal plasticity [7]. Additionally, α-synuclein is involved in the formation of the soluble N-ethylmaleimide-sensitive factor attachment protein receptor (SNARE) complex at presynaptic nerve terminals. This latter function is thought to be mediated by a chaperone activity that has not yet been identified, highlighting α-synuclein’s potential role in ensuring efficient neurotransmission and synaptic function [16,21].

### 2.2. Role of α-Synuclein in PD

**Figure 2:**
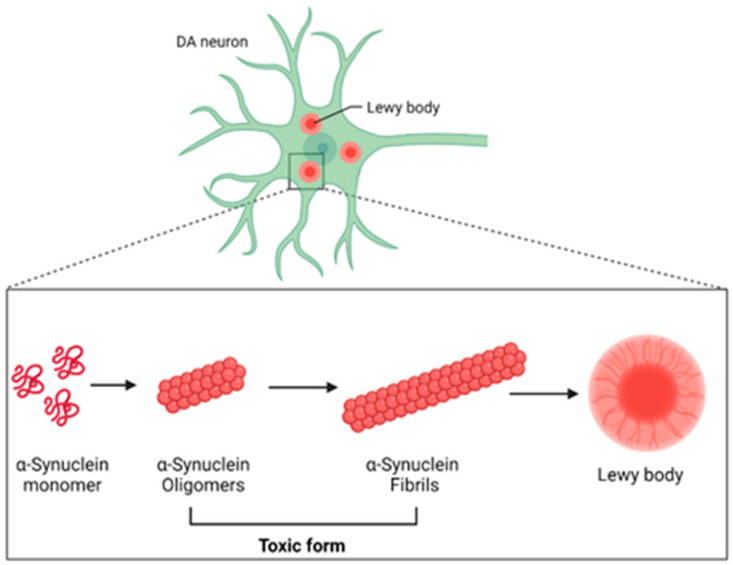
The role of α-synuclein in the pathogenesis of PD. DA: dopaminergic (created with BioRender.com on 15 July 2023).

α-synuclein plays a significant role in the pathogenesis of both familial and sporadic forms of PD [17], as well as in other synucleinopathies [22]. Under normal conditions, native α-synuclein exists in a dynamic equilibrium between unfolded monomers and α-helically folded tetramers with a low propensity for aggregation [23]. The aggregation process of α-synuclein involves a conformational change whereby it adopts a β-sheet-rich structure that facilitates its aggregation into oligomers, protofibrils, and insoluble fibrils that finally accumulate in Lewy bodies within neurons [24]. Almost all pathologically aggregated α-synuclein is phosphorylated at the Ser129 site [25]. These aggregates disrupt various cellular processes, leading to neuronal dysfunction and, ultimately, cell death [26,27] (Figure 2).

One of the critical ways that misfolded α-synuclein contributes to neuronal dysfunction is through the disruption of mitochondrial function [28,29,30]. Mitochondria are essential for energy production, and their dysfunction leads to reduced cellular energy levels, increased oxidative stress, and the activation of apoptotic pathways [31,32]. Misfolded α-synuclein aggregates can impair mitochondrial dynamics, including fission and fusion processes, and interfere with mitochondrial transport along axons, further exacerbating neuronal dysfunction [33].

Another significant impact of misfolded α-synuclein is on synaptic function [34]. Synapses are critical for neuronal communication; therefore, any disruption in their function can lead to significant impairments in neuronal signaling [35]. Misfolded α-synuclein can impair synaptic vesicle release and reuptake, leading to reduced neurotransmitter availability and synaptic transmission efficiency. This disruption contributes to the motor and cognitive symptoms observed in PD and related disorders [36,37].

### 2.3. α-Synuclein Propagation

Studies have suggested that α-synuclein can be transmitted between neurons [38] and can seed the formation of toxic aggregates in recipient neurons in a prion-like manner [39]. α-synuclein demonstrates several prion-like characteristics, notably the capacity of its aggregated forms to self-propagate by promoting the aggregation of normal α-synuclein, a process known as ‘seeding’. This mechanism can increase the aggregate burden within a cell. Similar to prions, misfolded α-synuclein can act as a template, inducing the misfolding of native α-synuclein in neighboring neurons. This templated misfolding can lead to the cell-to-cell transmission of pathological α-synuclein aggregates, contributing to the progressive nature of PD. For example, fetal dopamine cells transplanted into the striatum of patients with PD were found to develop Lewy pathology when examined neuropathologically 1–2 decades later [40]. Similar to the human transplants, α-synuclein has been observed in cell culture and rodent transplantation experiments, where it was transferred from one cell to another [38,41,42].

## 3. Gut–Brain Axis: A New Frontier in Parkinson’s Disease Research

The gut–brain axis represents a complex communication network linking the GI tract and the CNS, fundamentally impacting health and disease [43]. Research has highlighted that gut microbiota might play a significant role in the onset and progression of PD [44]. Various studies indicate that alterations in the gut microbiome have been observed in PD patients, with changes in the abundance of certain bacterial species compared to healthy controls. Additionally, α-synuclein also accumulates in the ENS [45,46,47]. This accumulation can occur years before the typical motor symptoms of PD appear, suggesting that the gut might be an early site of disease pathology. Understanding the gut–brain axis in PD could open new avenues for early diagnosis and targeted treatments that modulate the gut microbiota or its metabolic outputs, potentially slowing disease progression or alleviating symptoms.

### 3.1. Gut–Brain Axis

The concept of the gut–brain axis describes bidirectional communication between the CNS and ENS of the GI tract, which is linked by neurons of the sympathetic and parasympathetic nervous system [48,49]. Through this bidirectional communication network, signals from the brain can influence the motor, sensory, and secretory modalities of the GI tract and, conversely, visceral messages from the GI tract can influence brain function [50]. At present, it is suggested that gut microbes may serve as significant contributors to the bidirectional communication that takes place along the gut–brain axis. The microbiome community carries out important metabolic and physiological functions for the host and contributes to overall health and homeostasis [51,52,53]. Consequently, the gut microbiota has emerged as a potential diagnostic and therapeutic target in disorders as diverse as PD [54,55,56,57].

### 3.2. Dysbiosis of Microbiome in PD

Dysbiosis of the gut microbiome in PD patients is currently being investigated to determine which microbiota actively produce metabolites that are implemented in microglia activation, α-synuclein aggregation, and inflammation in the gut. Gut microbes play a crucial role in the gut–brain axis, which interacts with the ENS, enterocytes, and immune system to influence host health [58]. Evidence suggests that PD pathogenesis may be influenced by the interplay between the imbalance of gut microbes and altered bacterial metabolites [59,60,61]. Numerous findings in both observational PD patient studies and experimental animal studies have revealed that gut bacteria aid in the regulation of anti-inflammatory and pro-inflammatory profiles, suggesting that alterations within an individual’s gut microbiome can influence the risk of developing PD [44,57,62,63,64,65,66,67,68,69,70]. Newly diagnosed PD patients often exhibit intestinal hyperpermeability, endotoxemia, and microbial dysbiosis, which bolsters the hypothesis that gut-derived inflammation promotes neuroinflammation and neurodegenerative changes in PD pathogenesis [65,68,70,71]. Clinical studies have shown an increase in intestinal permeability in PD, where bacterial endotoxins in the form of lipopolysaccharides are associated with increased α-synuclein accumulation within the GI tract [72,73]. Some PD patients have shown elevated lipopolysaccharide serum levels, which could correlate to increased intestinal permeability [74,75,76]. Further, the guts of PD patients are often colonized by lipopolysaccharide-producing bacteria such as *Helicobacter pylori*, which induces chronic inflammation and degradation of the gut mucosal lining [9,75,77,78].

Individuals with high-risk factors for PD or the diagnosis of PD have been documented to have significantly different compositions of gut microbes compared to those of healthy controls [13,79,80,81]. The relative abundances of anti-inflammatory bacteria such as *Blautia*, *Coprococcus*, *Roseburia*, *Fusicatenibacter*, *Faecalibacterium*, and *Lachnospira* are reduced in PD patients, while, in contrast, *Lactobacillus*, *Bifidobacterium*, and *Akkermansia* phyla are higher in PD patients [65,76,82,83,84,85,86]. Numerous studies have documented the alterations in the composition of the gut microbiome in PD, as seen in Table 1 [44,68,82,83,87,88,89,90,91,92,93,94,95,96,97,98,99,100,101,102,103,104,105,106].

Lower levels of fecal SCFAs have been observed in PD patients compared to healthy controls [82,87,107,108], and the evidence suggests that SCFA-producing bacteria may modify the microbiome’s genetic potential to produce the enzymes needed for SCFA formation. A recent meta-analysis determined that PD patients had reduced levels of bacteria in the Lachnospiraceae and Ruminococcaceae families [65]. Similarly, another meta-analysis reported that PD patients had decreased levels of the genera *Roseburia* and *Faecalibacterium*, both belonging to the Firmicutes Phyla [83]. These bacteria are known SCFA producers, and reductions in their levels have been documented in other diseases with an inflammatory component. It is important to note, however, that not all studies have obtained the same results. Although it is not clear whether alterations in the microbiome are the origin of PD pathogenesis, it is evident that disease pathology influences the microbiota over time and vice versa.

### 3.3. Gut–Brain Axis and α-Synuclein

α-synuclein is a protein primarily found in the brain; however, recent evidence suggests that it also occurs in other peripheral tissues such as the GI tract [109,110,111]. Biopsies of GI tissues from PD patients have shown α-synuclein accumulation in the lower parts of the esophagus, stomach, duodenum, colon, and rectum [112,113,114]. Finding α-synuclein outside the CNS supports the hypothesis that the presence of α-synuclein in both the brain and the gut may result from a common pathological aggregation pathway involving the vagus nerve. This evidence indicates that the initial process of α-synuclein misfolding and pathology might start in the GI tract before spreading to the brain [115,116]. This observation supports Braak’s hypothesis, which proposes that α-synuclein aggregates originate in the gut and then propagate through the gut–brain axis, triggering the onset of PD [117,118]. Further, the retrograde spread of α-synuclein towards the brain suggests that GI-derived α-synuclein could ascend the ENS and propagate through interconnected neurons toward the midbrain of the CNS via the vagus nerve, ultimately reaching the Substantia nigra pars compacta (SNpc) and forming fibril structures that cause motor dysfunction [47,116,117,119,120].

Research suggests that certain microbes may influence the misfolding and abnormal formation of α-synuclein through extracellular mechanisms [121,122,123,124]; an example of this may be *Helicobacter pylori*, a gram-negative spiral bacterium known as the causative agent of gastric ulcers, present in 50% of the world population [125]. *H. pylori* contains cholesterol-α-glucosyltransferase, which catalyzes the conversion of membrane cholesterol to cholesteryl glucosides. The glycosylated derivatives can exert a neurotoxic effect on dopamine neurons and promote the aggregation of α-synuclein in the vagus nerve from the level of the stomach [126,127]. Another gram-negative bacteria species, i.e., *Escherichia coli,* has been documented to be implicated in α-synuclein misfolding [128]. *E. coli* produces an amyloid fiber protein called curli, whose purpose is to promote cell community behavior through the formation of biofilms in the extracellular matrix. The administration of *E. coli* in animal models has demonstrated an increase in α-synuclein fibril reactivity and accumulation of insoluble α-synuclein in the substantia nigra portion of the midbrain compared to controls, indicating that exposure to microbial amyloids in the GI may accelerate α-synuclein aggregation in the gut and brain [129,130].

#### 3.3.1. Braak’s Hypothesis

Braak’s hypothesis is a significant theory in the field of neurology, particularly concerning the pathology of PD. Proposed by Braak and his colleagues in the early 2000s, this hypothesis suggests that PD may start outside the brain, specifically in the gut or the nasal cavity, and then spread to the brain via the nervous system. According to Braak’s hypothesis, an unknown pathogen (either a virus or bacterium) in the gut could trigger the onset of sporadic PD [117]. Along with this hypothesis, they presented a staging system for PD that is based on a specific pattern of the spread of α-synuclein [131]. Subsequent to these publications, the broader dual-hit hypothesis was proposed, which posits that sporadic PD begins simultaneously in two locations: the neurons of the nasal cavity and the neurons in the gut [127,132]. From these places, the pathology is hypothesized to spread according to a specific pattern, i.e., via the olfactory tract and the vagus nerve, respectively, toward and within the CNS. There is experimental and clinical evidence supporting Braak’s hypothesis [133,134,135,136,137,138,139,140,141]. Additionally, α-synuclein aggregations have been found in the GI tract of animal models of early and advanced PD [142,143,144,145].

Braak’s hypothesis has had a substantial impact on research directions, focusing attention on the potential role of the gut–brain axis and the olfactory system in the early detection and understanding of PD [146,147,148]. It also supports the observation that non-motor symptoms, such as the loss of smell or GI issues, can precede the motor symptoms by several years.

#### 3.3.2. α-Synuclein from the Gut to the Brain

The transmission of α-synuclein from the ENS to the CNS is a critical research area for understanding the pathogenesis of neurodegenerative diseases, particularly PD. This area has gained significant attention as it may explain the initiation and progression of PD [12,149,150].

Direct evidence of gut–brain α-synuclein transmission in rodents was provided by Holmqvist et al. [47] who demonstrated that α-synuclein fibrils derived from PD patients could migrate from the GI tract to the brain via the vagus nerve in rats. Challis et al. [151] complemented these findings by inoculating the duodenal walls of aged mice with α-synuclein pre-formed fibrils (PFFs) and observed the progression of α-synuclein pathology to the brain. Similarly, Kim et al. provided additional evidence by injecting mouse α-synuclein PFFs into the pylorus and duodenum, noting the subsequent detection of phosphorylated α-synuclein in the CNS, beginning in the dorsal motor nucleus of the vagus nerve and progressing to regions like the locus coeruleus, amygdala, substantia nigra, and prefrontal cortex [119]. This progression closely reflects the Braak staging scheme for PD. Crucially, the study showed that these effects were negated if α-synuclein PFFs were injected into the intestinal wall following truncal vagotomy, which involves the severing of the vagus nerve. In this experimental setup, phosphorylated α-synuclein was still detectable in the upper duodenum 7 months after injection, but there was no spread to the substantia nigra [119]. These findings robustly support the notion that gut-derived α-synuclein is capable of propagating through the vagus nerve in a prion-like manner to induce CNS disease.

## 4. Polyphenols: Beyond Antioxidants

### 4.1. Types and Dietary Sources

**Figure 3:**
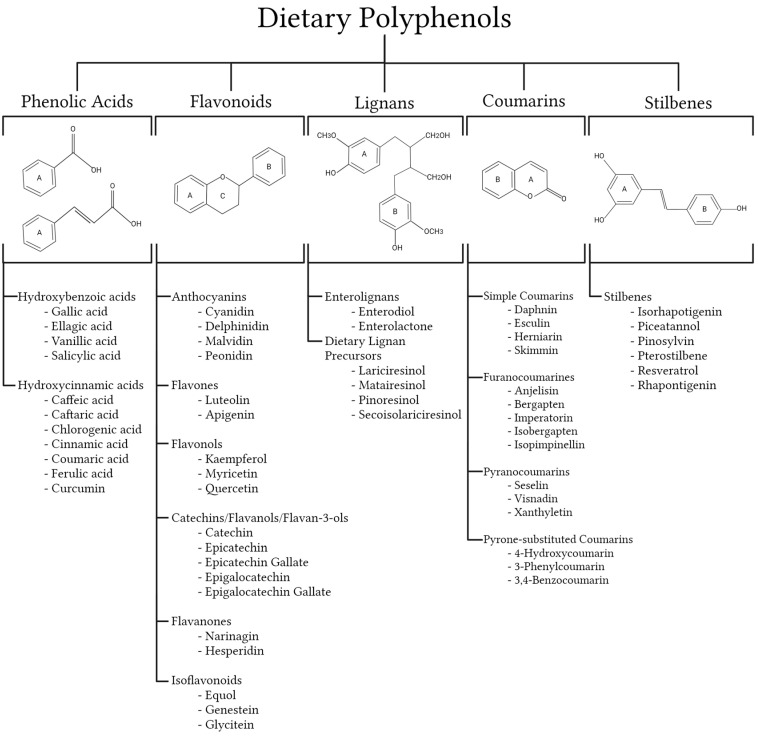
Classification of major dietary polyphenols (created with https://www.biorender.com/, accessed on 17 October 2023).

Polyphenols are highly specific secondary metabolites of plants that have a variety of biological roles, from resistance to infection by pathogenic microorganisms to defense against ultraviolet radiation [152]. Generally speaking, “polyphenol” refers to compounds derived entirely from the shikimate/phenylpropanoid pathway and/or the polyketide pathway, characterized by the presence of more than one phenolic unit and devoid of nitrogen-based functions [153]. To date, thousands of polyphenols have been identified from natural dietary plants. In general, polyphenols are mainly classified as phenolic acids, flavonoids, lignans, coumarins, and stilbenes, as outlined in Figure 3 [154]. The presence of polyphenol compounds in food greatly depends on environmental factors and food processing, production, and storage [152].

#### 4.1.1. Phenolic Acids

Phenolic acids are phytochemicals found in the majority of plant tissues and comprise a phenolic ring with an attached organic carboxylic acid. The secondary plant metabolites are aromatic acids that can be further classified into two broad groups, including hydroxycinnamic acids (HCAs) and hydroxybenzoic acids (HBAs), the latter of which are less common. HCAs are most concentrated in the outer ripened parts of fruits and are present in a variety of fruits, vegetables, and seeds [155,156]. The most abundant HCAs include the free forms of para-coumaric acid, caffeine, ferulic acid, and sinapic acid, whereas the bound forms include glycosylated derivatives of quinic acid, shikimic acid, and tartaric acid [157]. The most common HBAs include gallic acid, ellagic acid, syringic acid, and salicylic acid [158]. A wide variety of fruits and vegetables contain phenolic acids, including apples that contain chlorogenic acid and blueberries that contain para-coumaric, caffeic, and ferulic acids [159]. Phenolic acids are potent antioxidants due to their high potential to serve as hydrogen donors and oxygen quenchers and reduce metal-chelating agents [156,160,161]. Furthermore, HCAs and HBAs have been found to exhibit protective effects on neural, cardiac, and hepatic tissues, making this group of polyphenols an interesting target for research in the field of therapeutic medicine.

#### 4.1.2. Coumarins

Coumarins belong to the benzopyrone family and comprise a six-membered benzene ring attached to an α-pyrone ring [162]. Coumarins are present in several plants but are highest in concentration in the tonka bean, vanilla grass, sweet clover, and cinnamon [163]. These small molecule candidates have been considered for drug development due to their high solubility, low molecular weight, low toxicity, and high bioavailability [163,164]. Coumarins act as secondary plant metabolites to protect against herbivores and microorganisms through interactions with plant growth hormones and respiration; however, regarding biochemistry and physiology, coumarins are known to exhibit antioxidant effects and act as enzyme inhibitors [165]. Coumarins can be further classified into four groups: simple coumarins, furanocoumarins, pyranocoumarins, and pyrone-substituted coumarins, whereas both the free and glycosidic forms of coumarin can be found in plants [166]. While coumarins pose as promising therapeutic agents for human health, there are certain derivatives of coumarins, such as coumarin chromen-2-one, which are potentially poisonous for human consumption [167]. Still, the majority of coumarins have been recognized as harmless compounds and are considered to be candidates for medicinal drugs with diverse pharmacological activities, high bioavailability, and generally low toxicity [165].

#### 4.1.3. Stilbenes

Stilbenes accumulate in the vine tissues of plants under stressful biotic and abiotic conditions [168]. Over 400 natural stilbenes have been described [169], and their potential as antioxidants and chemopreventive agents is of interest to the scientific community. A major stilbene of interest, namely, resveratrol, has been researched for its potential cardioprotective effects. Resveratrol has been identified in at least 185 different plant species and is associated with antioxidant activity and its positive effect on lifespan and age-related diseases [170,171]. The regular consumption of stilbenes has been demonstrated to alleviate intracellular oxidative stress, reduce chronic inflammation, and suppress adipogenesis and lipogenesis [172].

#### 4.1.4. Lignans

Lignans consist of two phenylpropanoid units coupled by a β-β′-bond and are found in various seeds, grains, vegetables, and fruits [173]. Lignan bioavailability depends heavily on the diet due to its relatively low concentrations [174]. As a polyphenol class, lignans have received growing attention given their potentially beneficial bioactive properties due to their steroid-analogous chemical structure. Technically considered phytoestrogens, lignans have been documented to exhibit anti-estrogenic, antioxidant, and anti-carcinogenic effects. The lignans most common in the human diet include lariciresinol, matairesinol, pinoresinol, and secoisolariciresional which are most concentrated in sesame and flax seeds [173,175,176]. While the database on the content of lignans in foods is growing, more evidence needs to be added on the number of lignan dietary sources and the physiological role that the diverse compounds play in vivo.

#### 4.1.5. Flavonoids

Flavonoids are the largest class of dietary polyphenols and, hence, contribute greatly to our understanding of the broad spectrum of their health-promoting effects. There are over 6000 flavonoids that contribute to the pigments and protection of different fruits, vegetables, and medicinal plants [152]. All flavonoids contain at least 15 carbons with two benzene A and B rings and are further classified based on variations within the heterocyclic C ring [158]. The main groups of flavonoids are flavones, flavonols, catechins, isoflavones, flavanones, and anthocyanins. A broad spectrum of health-promoting effects has been attributed to the flavonoid group largely due to their ability to modulate key cellular enzyme functions and act as antioxidant, anti-inflammatory, anti-mutagenic, and anti-carcinogenic compounds [177]. Research has shown that flavones and catechins have the highest biological significance as antioxidants for protection against reactive oxygen species (ROS). Further, flavonoids are capable of inhibiting enzymes such as phosphodiesterase, lipoxygenase, Ca^2+^ ATPase, and COX, which are implicated in neurodegenerative diseases [177]. Researchers have also recognized flavonoids for their antimicrobial [178,179], antifungal [180], and antiviral [181,182] activities in vivo.

### 4.2. Gut Microbiota and Polyphenols

Dietary polyphenols can interfere directly with the enhancement or impairment of nutrient absorption. Given the antioxidant capabilities of many polyphenols, macro- and micronutrient oxidation may be prevented during metabolism, which could protect the quality of the nutrients. Carbohydrate absorption and glycemia may be decreased by polyphenols through their amylase-antagonizing activities [183,184], providing carbohydrates for bacteria such as *Bacteroides*, *Bifidobacterium*, *Clostridium*, *Eubacterium*, *Lactobacillus*, and *Ruminococcus* [185]. In this sense, polyphenols can influence bacterial diversity, gut peptide synthesis, energy and nutrient absorption, and insulin sensitivity [158]. However, polyphenol’s antioxidant capabilities may be a double-edged sword as polyphenols can interfere with mineral absorption. Gallic acid, chlorogenic acid, and polyphenolic polymerization products have been demonstrated to inhibit iron absorption [186,187,188,189]. Tannins and gallic acid have been documented to have an affinity for binding to zinc, further impairing zinc absorption [190,191]. Regardless of the caveats that accompany dietary polyphenols, research has elucidated the beneficial potentials and relationships between the gut microbiome and commonly consumed polyphenol substances.

#### 4.2.1. Curcumin

Curcumin, commonly known as turmeric, is a popular HCA that is known for its variety of pharmacological and restorative effects, including therapeutic approaches for chronic diseases, cardiovascular disease, depression, skin diseases, obesity, diabetes, and multiple types of cancer [192,193,194,195]. Studies that have observed the pharmacokinetics and bioavailability of curcumin have documented how poorly absorbed and rapidly metabolized the compound is, resulting in speculation about its clinical use [196]. Nevertheless, peripheral influences of curcumin may play a more important role in the CNS [197]. Curcumin may prevent the formation of ROS and glial cell activation, further hindering α-synuclein aggregation and neuronal cell apoptosis [197,198,199,200]. It has been proposed that curcumin can modulate gut signaling pathways and potentially exert effects on the microbiome gut–brain axis. Oral administration of curcumin has been demonstrated to promote beneficial gut bacteria growth, including *Bifidobacteria* and *Lactobacilli* strains, while reducing the abundance of pathogenic bacteria strains, including *Prevotellaceae*, *Coriobacterales*, *Enterobacteria*, and *Rikenellaceae* [201,202,203]. The primary metabolite of curcumin, i.e., tetrahydrocurcumin, has been documented to restore gut microbiome dysbiosis by lowering the relative abundance of *Actinobacteria* and *Proteobacteria* and by modifying the ratio of *Firmicutes* to *Bacteroidetes* [204]. An in vivo study showed that curcumin intervention improved motor deficits, glial cell activation, and the aggregation of α-synuclein in a 1-methyl-4-phenyl-1,2,3,6-tetrahydropyridine (MPTP) PD mouse model. The microbiome data showed elevated levels of *Lactobacillaceae*, *Lachnospiraceae*, *Muribaculaceae*, and *Eggerthellaceae* in the treatment groups compared to controls [197]. These studies suggest that oral administration of curcumin may promote the growth and proliferation of beneficial gut microbes.

#### 4.2.2. Anthocyanins

Anthocyanins are water-soluble pigments in the polyphenol family, found in flowers and fruits that have red, blue, or purple hues as well as in nuts and certain vegetables. Anthocyanins are a group of flavonoids with a wide range of uses, such as medicinal herbs, including antidiabetic, anticancer, anti-inflammatory, antimicrobial, and anti-obesity effects [205]. The most common types of anthocyanins include cyanidin, delphinidin, pelargonidin, peonidin, petunidin, and malvidin, which are all potent nutraceutical or pharmaceutical components. These compounds have low bioavailability due to their rapid absorption in the stomach and small intestine, causing low absorption into the blood and circulatory system and high excretion rates and reducing the efficiency of anthocyanins as free-radical scavengers. An in vitro incubation study in 2012 assessed anthocyanins’ effects on the growth of *Bifidobacterium* spp., *Lactobacillus* spp., and *Bacteroides* spp. and found that the mixture of malvidin-3-glucoside, delphinidin-3-glucoside, peonidin-3-glucoside, petunidin-3-glucoside, and cyanidin-3-glucoside had a synergistic effect on the growth of beneficial bacteria [206]. One of the microbiota metabolites of anthocyanin, namely, gallic acid, was observed to have an inhibitory effect on a group of potentially harmful bacteria, including *Clostridium histolyticum*, without affecting the beneficial bacteria. Furthermore, gallic acid produced from the administration of the anthocyanin mixture significantly reduced the *Bacteroides* spp. group [206]. A 2012 intervention study aimed to evaluate the effect of the moderate intake of anthocyanins in red wine on certain gut microbial groups on host health benefits and found that red wine polyphenols could have a significant effect on the growth of select gut microbes, including *Proteobacteria*, *Fusobacteria*, *Firmicutes*, and *Bacteroidetes* [207].

#### 4.2.3. Tea Polyphenols

The evergreen shrub *Camellia sinesis* can be consumed as unfermented green tea, semifermented oolong tea, and fermented (oxidized) black tea [208,209]. Tea leaves contain a variety of components, including polyphenols, terpenoids, purine alkaloids, the amino acid L-theanine, carbohydrates, caffeine, theaflavins, and a mixture of natural flavonoids called tea catechins (TCs). The health benefits of tea have been attributed in part to their antioxidant and anti-inflammatory properties; however, some benefits have been linked to the relationship between tea bioactive compounds and the gut microbiota [210]. Tea polyphenols have a low bioavailability; additionally, it has been estimated that 90–95% of the dietary polyphenol compounds travel to the large intestine where they are in direct contact with the gut microbiota [211]. Both green and black teas are rich in flavonoids, comprising up to 30% of their dried volume [212].

##### Theaflavins

Theaflavin (3,4,5-trihydroxybenzocyclohepten-6-one) is a polyphenol and biflavonoid responsible for the red pigment in black and oolong tea formed by oxidation during fermentation [213,214]. Theaflavins (TFs) are further divided into theaflavin (TF1), theaflavin-3-gallated (TF2A), theaflavin-3′-gallate (TF2B), and theaflavin-3,3′-digallate (TF3) [215]. TFs consist of a benzotropolone skeleton that is formed by the oxidation of epicatechin (EC) and epigallocatechin-3-gallate (EGCG) in the presence of polyphenol oxidase and peroxidase enzymes [216]. TFs contribute to the health-promoting effects of the gut microbiota due to the bacteria-mediated metabolism that occurs in the lower GI tract. TFs that reach the large intestine are modified by ring-cleavage, reduction, hydrolysis, decarboxylation, and dihydroxylation reactions [217,218]. An in vitro fecal fermentation study demonstrated that TF administration promoted the growth of *Bacteroides*, *Lachnoclostridium*, *Faecalibacterium*, *Parabacteroides*, and *Bifidobacterium*, whereas *Prevotella* and *Fusobacterium* growth was inhibited [219]. TF3 was shown to inhibit the growth of gram-negative bacteria, including *Klebsiella aerogenes*, *Escherichia coli*, *Pseudomonas aeruginosa*, and *Proteus mirabilis,* and gram-positive bacteria, including *Staphylococcus aureus*, *Streptococcus pyogenes*, and *Mycobacterium smegmatis* [220].

The exerted health benefits may be due to the regulation of various cellular signaling pathways, including signaling to mitigate cellular inflammatory responses and blocking of the mitogen-activated protein kinase [221]. TFs have several therapeutic properties, including a protective effect on neuronal cell damage, promotion of immune response, and protection against certain disease initiation via the induction of apoptosis, cell cycle arrest, and suppression of inflammation [222,223]. TFs have been documented to act as strong free-radical scavengers, inhibit oxygen radical-mediated lipid peroxidation, and induce activation in different antioxidant enzymes [215,224]. TFs have been demonstrated in vivo to have the ability to penetrate the blood–brain barrier (BBB) and offer neuroprotection via radical scavenging, antioxidative, antiapoptotic, and cell-regulating pathways [222,225,226]. When TFs are hydrolyzed by *Bifidobacteria* and *Lactobacilli* species, the corresponding metabolites TF1, TF2A, TF2B, gallic acid, and pyrogallol become available metabolites in circulation [218,227]. Additionally, two microbial metabolites of TFs, i.e., 3-4′-hydroxyphenylpropionic acid and gallic acid, can increase tyrosine hydroxylase (TH) and dopamine transporter (DAT) immune responses. TFs should be considered promising prebiotic components due to their capacity to increase the abundance of beneficial bacteria and decrease the abundance of potentially harmful bacteria.

##### Green Tea Catechins

There are four major TC compounds found in green tea leaves, identified as (-)-epicatechin (EC), (-)-epigallocatechin (EGC), (-)-epicatechin gallate (ECG), and (-)-epigallocatechin gallate (EGCG) [228], whereas EGCG is the most abundant and the most active component among catechins. TCs offer a spectrum of health benefits to animals and humans, including protection from cardiovascular diseases [229], cancers [230], and neurodegenerative diseases [231,232]. Several cell-based and animal experiments have provided evidence demonstrating that tea polyphenols and TCs have beneficial effects against PD [66,209,233,234,235]. The mode of action of TCs is mainly through the prevention of aggregate formation and dopamine loss, alleviating mitochondrial dysfunction, antioxidative stress, and anti-neuroinflammation, as well as the activation of neurotrophic factor and signaling pathways [236]. Metabolites of TCs are readily absorbed into circulation and may lead to the enhanced bioavailability and bioactivity of TCs [237,238,239]. TCs have been documented to modify gut-microbiota-dependent metabolisms of bile constituents and micronutrients [240], including the TCA cycle, bile acid metabolism, and metabolism of purine, pyrimidine, and amino acids [241]. EGCG has been reported to cross the BBB after ingestion, leading to increased activity of antioxidants, iron chelation, and antimutagenic effects [232]. Additionally, aromatic rings and hydroxyl groups produced by TC metabolism may provide neuroprotection by reducing lipid peroxidation and antioxidant activity [242].

Overall, the evidence has demonstrated that TCs can significantly impact the biodiversity of host gut microbiota and overall health by stimulating or hindering the growth of certain microbial species [210,243]. Previous studies [234] have shown that long-term supplementation with TCs significantly affected the compositions of the gut microbiome in time-dependent and dose-dependent patterns in Sprague–Dawley (SD) rats. *Bacteroidetes* and *Oscillospira*, both beneficial microbial families, were significantly enriched, while *Peptostreptococcaceae*, a gram-positive bacterium that is overrepresented in the gut of colon cancer patients, was significantly diminished. TCs have shown inhibitory effects on certain bacteria, such as *Bacillus cereus*, *Campylobacter jejuni*, *Clostridium perfringens*, *E. coli*, *H. pylori*, *Legionella pneumophila*, and *Mycobacterium* species [244]. Gram-negative bacteria are surrounded by a negatively charged lipopolysaccharide membrane that repels catechins [245]; however, some evidence shows that TC supplementation can inhibit the growth of harmful gram-negative bacteria. A 2013 study [246] found that in vitro EGCG, gallocatechin gallate (GCG), and EGCG 3”-methyl could significantly increase the abundance of beneficial bacteria, including *Bifidobacterium* spp., *Lactobacillus*, and *Enterococcus*, successively increasing the production of SCFAs. It was also observed that catechins were able to reduce the growth of *Bacteroides*, *Prevotella*, *Clostridium hystoliticum*, *Eubacterium*, and *Clostridium* species [246].

### 4.3. Polyphenols and α-Synuclein

Research has demonstrated that α-synuclein is a crucial therapeutic target for PD, and inhibiting its aggregation, oligomerization, and fibrillation are key strategies for disease modification [247,248]. Numerous studies have identified a variety of polyphenolic compounds capable of inhibiting fibrils and oligomer formation, as well as stabilizing or disaggregating the α-synuclein oligomers [249,250,251,252]. This makes them promising therapeutic candidates for PD and related synucleinopathies. EGCG, quercetin, polyphenolic acids, curcumin, and their derivatives are some of the most well-known and effective polyphenolic inhibitors of α-synuclein.

#### 4.3.1. EGCG

EGCG is a well-studied inhibitor of α-synuclein fibrillization, with a notable inhibition concentration (IC50) of 9.8 μM [253,254,255,256]. First reported by Ehrnhoefer in 2008 [253], EGCG inhibits α-synuclein by redirecting its aggregation into stable, spherical, and off-pathway oligomers and transforming pre-formed fibrils into unstructured aggregates. Apart from redirecting the aggregated α-synuclein to off-pathway oligomers, EGCG was reported to be able to transform already-formed fibrils into unstructured aggregates without releasing monomers [257]. The binding of EGCG with α-synuclein is non-specific, and it exhibits anti-amyloidogenic properties against multiple proteins, including Aβ and huntingtin [258,259,260]. Further studies suggest that EGCG crosslinks α-synuclein into compact structures that cannot bind to the normal fibrils [261]. Importantly, EGCG was reported to bind to the same binding sites on α-synuclein fibrils as Thioflavin T(ThT) and substitute it, leading to misinterpretation of the aggregation readout [262]. Its efficacy, including in cells overexpressing α-synuclein or its A53T mutant [256], suggests that EGCG-induced aggregates are less likely to damage cell membranes compared to aggregates formed in its absence [263]. However, the full scope of EGCG’s activity remains under investigation and is not yet fully understood [264].

#### 4.3.2. Curcumin

Curcumin has been recognized for its potential to slow the progression of neurodegenerative diseases such as PD [265]. Over the past few decades, the effects of curcumin on α-synuclein aggregation have been widely studied [266,267,268,269,270,271,272,273]. It has been reported that curcumin can inhibit α-synuclein fibrillization in vitro and disassemble already-formed fibrils [266,267,270]. Additionally, several modified analogs of curcumin with improved stability have also been proven effective in inhibiting α-synuclein amyloid aggregation and depolymerizing α-synuclein fibrils [269,274]. Interestingly, some studies suggested that curcumin may bind to oligomers and fibrils, thereby accelerating α-synuclein fibrillation, while producing less toxic α-synuclein aggregates [266,275]. Curcumin has also been shown to prevent hexokinase I (HKI) release and reactive oxygen species (ROS) enhancement triggered by α-synuclein fibrils in mitochondria [276,277]. Animal testing in an α-synuclein transgenic PD mouse model has demonstrated that the curcumin-rich diet showed improvements in mice motor behavior, although no changes in aggregate levels were detected [278].

Although in vitro data have shown that many polyphenols successfully modify α-synuclein, they still require systematic pharmacokinetic evaluations through in vivo studies. Additionally, many polyphenolic compounds face challenges in crossing the BBB due to their non-lipophilic nature, potentially preventing them from reaching the concentrations necessary to exert effects in the brain [279,280]. Several factors, such as stability, solubility in an acidic environment at a gastric pH, absorption pattern, gut microbiota, enterohepatic circulation, first-pass metabolism, and metabolic fate concerning phase I or phase II metabolism, play a key role in achieving the ideal bioavailability of the phytochemicals in the brain [281,282].

## 5. Interplay between Polyphenols, Gut Microbiota, and α-Synuclein

**Figure 4:**
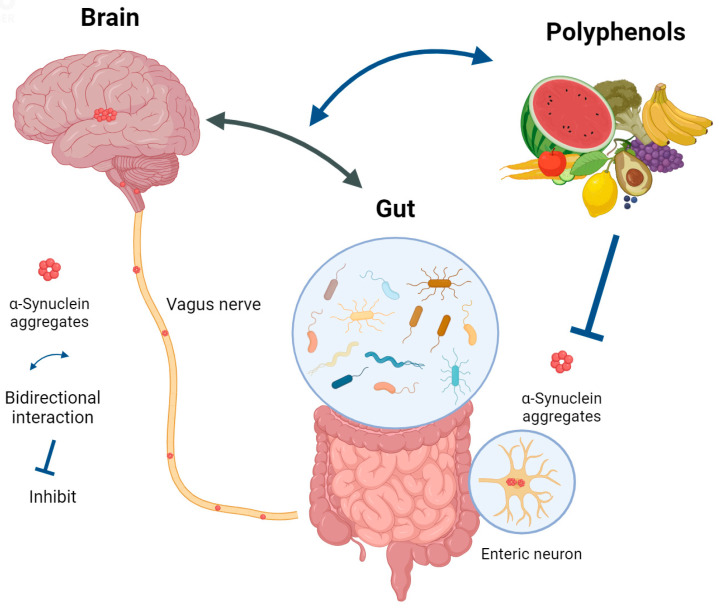
The interactions between polyphenols, the gut–brain axis, and α-synuclein (created with https://www.biorender.com, on 21 May 2024).

With increasing recognition of the importance of the gut–brain axis in PD etiology, there is increasing interest in developing interventional strategies that target and attenuate α-synuclein aggregation and dispersion. As discussed in the above section, polyphenols are capable of regulating the gut microbiome composition and the metabolic pathways, mostly through the inhibition of pathogenic bacteria and the stimulation of beneficial bacteria [283,284,285]; in turn, polyphenols are extensively metabolized by the gut bacteria, resulting in the generation of bioactive secondary metabolites, which enhance bioavailability [286]. These gut bacterial metabolites inhibit fibrils and oligomer formation and stabilize or disaggregate the α-synuclein oligomers. More recent research suggested that the microbial metabolites of polyphenols can inhibit the spread of α-synuclein in a cell-based system [249,287] (Figure 4). However, the mechanisms of such an interaction between polyphenols, gut microbiota, and α-synuclein are largely unknown.

### 5.1. Bioactivity and Efficacy of Polyphenols Are Affected by Gut Microbiota

Polyphenols that are most commonly found in dietary components are glycosides [288], which are not completely absorbed in the upper GI tract; only aglycones and some glucosides can be absorbed in the intestinal mucosa [289]. Recent evidence shows that the bioactivity and efficacy of polyphenols are significantly influenced by the gut microbiota [286,290,291,292]. The microbes in the gut can transform polyphenols into various metabolites that may have different bioactive properties from the original compounds [293,294]. Additionally, the composition of an individual’s gut microbiota, which varies greatly among individuals, can determine the degree to which polyphenols are metabolized and utilized [295]. For example, interpersonal heterogeneity in gut microbiota may lead to interpersonal variabilities for their efficacy in metabolizing dietary flavanols into certain biologically available phenolic acid metabolites that interfere with α-synuclein misfolding [252]. Preclinical investigations have demonstrated that dietary supplementation with select bioactive polyphenol-rich dietary preparations, such as a grape seed polyphenol extract (GSPE) and a standardized Bioactive Dietary Polyphenol Preparation (BDPP, comprised of a select Concord grape juice, GSPE, and resveratrol) is mechanistically effective in modulating diverse neuropathologic phenotypes [296]. Furthermore, treatment with a flavanol-rich preparation (FRP) in gnotobiotic mice yielded brain-bioavailable polyphenol metabolites [297]. A recent review highlighted that polyphenols’ neuroprotective effects can be direct, after the metabolites of polyphenols cross the BBB, or indirect, with influences on gut microbial communities [298]. Clinical trials have demonstrated that polyphenol metabolites are positively associated with cerebral blood flow and oxygenation, resulting in direct neuroprotective effects [299,300]. Meanwhile, the gut microbiome can produce active metabolites of polyphenols, which indirectly enhance their neuroprotective capacity, an example of which is curcumin being transformed into demethylcurcumin and bisdemethoxycurcumin [298,301]. Therefore, understanding the interaction between polyphenols and gut microbiota, and identifying the specific microbes that enhance the efficacy of polyphenols is crucial for developing therapeutic strategies and precision nutrition based on the microbiome.

### 5.2. Anti-α-Synuclein Microbial Polyphenol Metabolites

As α-synuclein is a crucial therapeutic target for PD, inhibiting its aggregation, oligomerization, and fibrillation are key strategies for disease modification [302]. Polyphenols are among the emerging therapeutic options for combatting abhorrent α-synuclein as they exist in a wide variety of chemical compounds and have the ability to produce secondary metabolites after undergoing microbial transformation by gut microbiota. These metabolites are known for their varied pharmacological properties, which have been extensively documented in scientific reviews [294,295,303,304].

Ono and colleagues [252] established two experimental groups of humanized gnotobiotic mice with diverse gut bacteria compositions and administered a polyphenol-rich preparation orally. They detected 15 polyphenol-derived microbial metabolites, which are generated by the gut microbiota fermentation of grape seed polyphenol extract, in the cecal compartment, including caffeic acid, ferulic acid (FA), gallic acid (GA), and vanillic acid at μM to sub-μM concentrations. Notably, three metabolites—3,4-dihydroxybenzoic acid (3,4-diHBA), 3-hydroxybenzoic acid (3-HBA), and 3-(3′-hydroxyphenyl) propionic acid (3-HPPA)—were found to accumulate in the brain. The in vitro study confirmed that 3-HBA, 3,4-diHBA, and 3-HPPA inhibit α-synuclein aggregation, including the formation of low-order oligomers such as dimers and trimers. The findings align with reports from another study that showed that 3-HBA and 3-HPPA prevent the misfolding and assembly of Aβ peptides into neurotoxic aggregates, such as Aβ oligomers [305]. Furthermore, using the A53T mutant *Drosophila* model of PD, these metabolites effectively improved motor dysfunction, indicating a mitigating effect on mutant α-synuclein-mediated neurotoxicity [306]. Very recently, 3-HPPA, 3,4-diHBA, 3-HBA, and 4-HBA were found to significantly attenuate intracellular α-synuclein seeding aggregation in a cell-based system, and the findings were confirmed using insoluble α-synuclein aggregates extracted from post-mortem Multiple System Atrophy (MSA) and PD brain specimens [249].

## 6. Conclusions and Perspectives

Dietary polyphenols are promising protective agents for PD prevention because of their abundance and relatively low toxicity [307]. Previous studies have shown that polyphenols can combat PD through multiple mechanisms, including reducing neuronal apoptosis, attenuating oxidative stress, and downregulating neuroinflammation. Recent evidence has also demonstrated that polyphenols inhibit the formation and spread of α-synuclein aggregates, a hallmark of PD pathology, which may originate from the gut. Furthermore, polyphenols are capable of regulating the gut microbiota composition and its metabolic pathways, mostly through the inhibition of pathogenic bacteria and the stimulation of beneficial bacteria [283]; in turn, polyphenols are extensively metabolized by gut bacteria, resulting in the generation of bioactive secondary metabolites that enhance their bioavailability.

While polyphenols showed benefits in animal models of PD, evidence from epidemiological studies primarily demonstrated the association between the dietary intake of polyphenols and a reduced risk of developing PD. However, data from prospective randomized controlled trials in patients with pre-existing PD are limited. Therefore, future clinical studies are necessary to evaluate the effectiveness of dietary polyphenols in slowing the progression of PD. Although the existing evidence points to the potential of polyphenols to favorably modulate the gut–brain axis, it is evident that more focused research is needed to fully understand the mechanisms between polyphenols, the gut microbiota, and α-synuclein in order to establish the therapeutic viability of polyphenols in clinical settings.

## Figures and Tables

**Table 1:** Altered gut microbes in patients with PD compared to healthy controls.

Phyla	Class	Order	Family	Genus	Species	Increased	Decreased
Bacteroidetes (Bacteroidota)	Phylum Bacteroidetes						87, 100, and 103
	Bacteroidia	Bacteroidales	Bacteroidaceae	*Bacteroides*		87 and 104	
				*Bacteroides*	*fragilis*		88
				*Bacteroides*	*coprocola*		93 and 106
			Barnesiellaceae			98	
			Odoribacteraceae	*Butyricimonas*		97	
				*Odoribacter*		97	
			Porphyromonadaceae			100	
				*Porphyromonas*		89 and 97	
			Prevotellaceae				44, 87, 94, 100, and 103
				*Prevotella*		89 and 90	82 and 97
				*Prevotella*	*copri*		91
			Rikenellaceae	*Alistipes*		106	
				*Alistipes*	*shahii*	91	
			Tannerellaceae			83 and 97	
				*Parabacteroides*		83 and 97	
Firmicutes (Bacillota)	Phylum Firmicutes					91 and 103	68, 102, and 104
	Erysipelotrichia	Erysipelotrichales	Coprobavillaceae	*Coprobacillus*		83 and 97	
			Erysipelotrichaceae	*Eubacterium*	*biforme*		91
					*hallii*		91
					*rectale*		91
					*eligens*		91
	Clostridia	Eubacteriales	Clostridiaceae	*Butyricicoccus*			89
				*Clostridium*	*saccharolyticum*		91
					*coccoides*		88
				*Hungatella*		83	
			Christensenellaceae	*Christensenella*		92, 93, 95, and 96	
				*Christensenella*	*minuta*	93	
			Carabacteraceae	*Catabacter*		83 and 93	
				*Catabacter*	*hongkongensis*	93	
			Lachnospiraceae				68, 83, 89, 92, 95, 96, 101, and 102
				*Agathobacter*			89
				*Blautia*			68, 89, and 99
				*Coprococcus*			68 and 83
				*Fusicatenibacter*			89
				*Lachnospira*			89
				*Roseburia*			68, 83, 89, 92, 95, and 96
			Oscillopiraceae (Ruminococcaceae)			100	
				*Faecalibacterium*			83, 89, 92, 95, and 96
				*Faecalibacterium*	*prausnitzii*		87
				*Hydrogenoanaerobacterium*		83 and 100	
				*Oscillospira*		68, 93, 95, 96, and 103	89
				*Ruminiclostridium*		83	
				*Ruminococcus*		95 and 100	
				*Ruminococcus*	*bromii*	93	
				*Papillibacter*	*cinnamivorans*	93	
	Bacilli	Lactobacillales	Lactobacillaceae			44, 83, 95, 98, and 101	87
				*Lactobacillus*		88, 89, 93, 96, 97, and 104	
				*Lactobacillus*	*mucosae*	93	
			Enterococcaceae			98 and 101	87
				*Enterococcus*		97 and 99	
			Streptococcaceae	*Streptococcus*		99	
	Negativicutes	Veillonellales	Veillonellaceae	*Veillonella*		97	
Proteobacteria (Pseudomonadota)	Deltaproteobacteria	Desulfovibrionales	Desulfovibrionaceae	*Bilophilia*		83, 92, 97, and 103	
				*Bilophila*	*wadsworthia*	92 and 105	
	Gammaproteobacteria	Enterobacterales	Enterobacteriaceae			44, 87, 94, 95, and 101	
		Pasteurellales	Pasteurellaceae			100	83
				*Haemophilus*			83
Actinobacteria (Actinomycetota)	Actinomycetia	Bifidobacteriales	Bifidobacteriaceae	*Bifidobacterium*		82, 87, 89, 92, 93, 95, 96, and 102	104
		Corynebacteriales	Corynebacteriaceae			95	
				*Corynebacterium*		89	
	Coriobacteriia	Coriobacteriales	Coriobacteriaceae	*Collinsella*		92, 95, and 103	
		Eggerthellales	Eggerthellaceae			83 and 103	
Verrucomicrobia	Phylum Verrucomicrobia					97, 100, 103, and 105	
	Verrucomicrobiae	Verrucomicrobiales	Akkermansiaceae	*Akkermansia*		68, 90, 92, 95, 96, 97, 100, 103, and 106	
				*Akkermansia*	*muciniphila*	87, 91, 105, and 106	
Synergistetes	Synergistia	Synergistales	Synergistaceae			83	
Deferribacteres	Deferribacteres	Deferribacterales	Mucispirillaceae	*Mucispirillum*		97	

## Data Availability

Not applicable.
